# Immune-system-dependent anti-tumor activity of a plant-derived polyphenol rich fraction in a melanoma mouse model

**DOI:** 10.1038/cddis.2016.134

**Published:** 2016-06-02

**Authors:** A Gomez-Cadena, C Urueña, K Prieto, A Martinez-Usatorre, A Donda, A Barreto, P Romero, S Fiorentino

**Affiliations:** 1 Grupo de Inmunobiología y Biología Celular, Pontificia Universidad Javeriana, Bogotá, Colombia; 2 Ludwig Cancer Research Center, University of Lausanne, Lausanne, Switzerland

## Abstract

Recent findings suggest that part of the anti-tumor effects of several chemotherapeutic agents require an intact immune system. This is in part due to the induction of immunogenic cell death. We have identified a gallotannin-rich fraction, obtained from *Caesalpinia spinosa* (P2Et) as an anti-tumor agent in both breast carcinoma and melanoma. Here, we report that P2Et treatment results in activation of caspase 3 and 9, mobilization of cytochrome c and externalization of annexin V in tumor cells, thus suggesting the induction of apoptosis. This was preceded by the onset of autophagy and the expression of immunogenic cell death markers. We further demonstrate that P2Et-treated tumor cells are highly immunogenic in vaccinated mice and induce immune system activation, clearly shown by the generation of interferon gamma (IFN-*γ*) producing tyrosine-related protein 2 antigen-specific CD8+ T cells. Moreover, the tumor protective effects of P2Et treatment were abolished in immunodeficient mice, and partially lost after CD4 and CD8 depletion, indicating that P2Et's anti-tumor activity is highly dependent on immune system and at least in part of T cells. Altogether, these results support the hypothesis that the gallotannin-rich fraction P2Et's anti-tumor effects are mediated to a great extent by the endogenous immune response following to the exposure to immunogenic dying tumor cells.

Melanoma as well as many other tumor types induce a strong immune response (IR) as evidenced in many cases by the presence of tumor infiltrating lymphocytes.^1^ However, the tumor immune-suppressive microenvironment limits T-cell effector activity contributing to tumor progression. The possibility to enhance tumor immunogenicity and induce an effective and protective response *in situ*, has been recently studied.^2^ It has been demonstrated that the use of agents rendering dying tumor cells visible to the immune system via the expression of ‘danger signals'^3, 4^ is a promising approach.

A new type of cell death called immunogenic cell death (ICD) has been described following some conventional anticancer chemotherapies such as anthracyclines.^5^ It has been shown that these chemotherapeutic drugs require an intact immune system to be effective against tumors. They elicit a tumor cell *in situ* vaccination inducing immune cells activation and an anti-tumor response. It has been proposed that three molecular signals provided by dying cells cooperate to render dead cells immunogenic. These are the calreticulin (CRT) exposure by cell membrane or ecto-CRT within the first hours of treatment during the early apoptotic stage;^6^ adenosine triphosphate (ATP) secretion during intermediate or late apoptosis^7, 8^ for which an intact autophagic machinery is needed,^9^ and finally high-mobility group box 1 (HMGB1) secretion during late apoptosis stage.^10, 11^

Plants used in the traditional Chinese medicine are known to significantly increase survival in patients with several types of cancer such as breast carcinoma,^12^ hepatocellular carcinoma,^13^ lung carcinoma^14^ or colon carcinoma.^15^ Some natural products are known to favor anti-tumor IR.^16^ For example genistein has been shown to increase the *ex vivo* cytotoxic activity of CD8 T cells in the P815 tumor model and to reduce the number of lung nodules in the B16F10 melanoma model.^17^ Furthermore, the epigallocatechin-3-gallate increases CD8 T-cell tumor infiltration^18^ and a plant extract from the Japanese traditional medicine called *Juzen-taho-to* was shown to induce a CD8 T-cell dependent anti-tumor IR in the Ret melanoma model.^19^

Recently, we obtained a gallotannin-rich standardized fraction (P2Et) from *Caesalpinia spinosa*. This fraction contains galloylquinic acid derivatives in high proportions as well as pentagalloylglucose and gallic acid-containing compounds (gallates) in lower proportions.^20^ In a previous study we showed that the P2Et fraction has anti-tumor activity in the 4T1 mammary mouse tumor model and decreases the number of lung metastasis.^21^

In this study, we show that treatment with P2Et, triggers *in vitro*, the exposure of CRT-charged vesicles by autophagic B16F10 cells and delays tumor growth, in an *in vivo* subcutaneous (s.c.) melanoma model. We further demonstrate that P2Et's anti-tumor activity is immune system dependent as it induces ICD, probably effective dendritic cells (DCs) activation and is associated with the enhanced generation of melanoma associated antigen-specific T cells.

## Results

### P2Et fraction induces apoptosis through caspase 3 and 9 activation of melanoma cells

**Figure 1:**
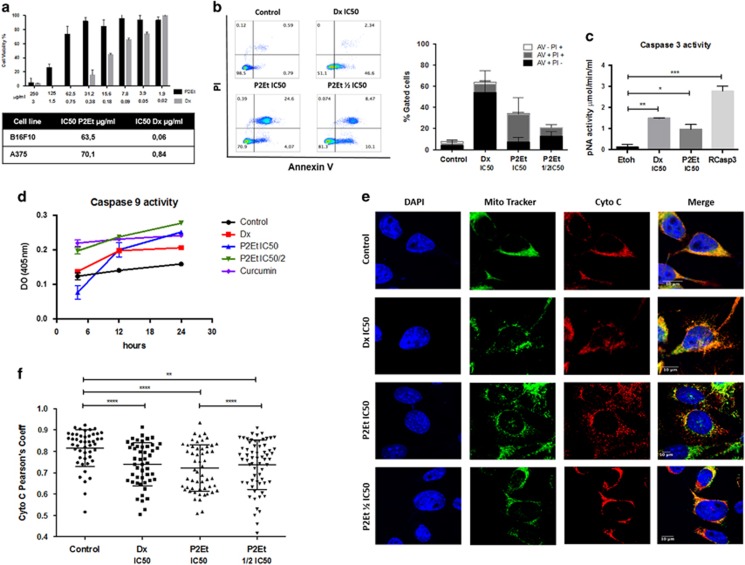
Treatment with P2Et fraction induces apoptosis in B16F10 melanoma cell line *in vitro*. (**a**) IC50: B16F10 cells were treated with various concentrations of P2Et, Dx or the respective negative controls (EtOH or DMSO) for 48 h and cell viability was determined by MTT assay, data was normalized to the viability of the controls (100%, treated with 0.5% DMSO or EtOH, vehicles). Results are mean values±S.E.M. (*n*=3). (**b**) Apoptosis analysis: it was made by Anexine V and PI assay, briefly cells were collected and stained 48 h after treatment and analyzed by flow cytometry. Results are mean values±S.E.M. (*n*=3). (**c** and **d**) Caspase 3 and 9 activity: the activities were evaluated by enzyme-linked immunosorbent assay, cells were treated for 48 h to evaluate caspase 3 and were we used recombinant caspase 3 as positive control (Rcasp3) and different time points for caspase 9 as indicated in the graph. Results are mean values±S.E.M. (*n*=3) *P<0.05; **P<0.01; ***P<0.001. (**e**) Cytochrome C mobilization. Cells were labeled with primary antibody anti-cytocrome c and detected with the conjugated anti-mouse secondary antibody (alexa fluor 647). Mitochondria were labeled with mitotracker Red TM and nuclei with DAPI. Mobilization was determined by confocal microscopy, after 24 h of treatment. Images were acquired with Olympus F1000 with a 60 × PlanAPO objective (*n*=2). (**f**) Person correlation coefficients: mitochondria and cytochrome c superposition was represented by Pearson's coefficient, each dot correspond to a single cell for a total of 50 cells per group (*n*=2) **P<0.01; ****P<0.0001

The P2Et fraction reduced viability of B16F10 and A375 in a dose-dependent manner (half maximal inhibitory concentration (IC50) of 63.5±12.5 *μ*g/ml on B16F10 and of 70.1±15.4 *μ*g/ml on A375 cells). Doxorubicin (Dx), was used as an ICD-positive control with an IC50 of 0.06 and 0.84 *μ*g/ml, respectively, as previously reported^22, 23, 24^ (Figure 1a). Moreover, we previously described the cytotoxic activity of P2Et on other cancer cells lines and its selectivity toward tumor cells. Low cytotoxicity with an IC50 >125 *μ*g/ml was reported for fibroblast and PMA/Ionomycin activated peripheral blood mononuclear cells.^21, 25^

Anti-tumor drugs inducing apoptosis through the mitochondrial intrinsic pathway are good candidates for cancer treatment.^26^ Therefore, we assessed whether P2Et treatment can induce intrinsic apoptosis in the B16F10 melanoma. We observed that B16F10 cells treated with P2Et showed high percentages of late apoptosis after 48 h (Figure 1b), but without higher percentages of necrotic B16F10 cells, suggesting that cell death was driven by apoptosis and not necrosis.^21^ Furthermore, we found caspase 3 (Figure 1c) and caspase 9 activation (Figure 1d), comparable to that observed with the positive controls Dx and curcumin.

To determine if P2Et-induced cell death involves the mitochondrial pathway, we evaluated cytochrome c localization within or outside the mitochondria. Images of B16F10 cells treated with either P2Et fraction or Dx showed cytochrome c mobilization to the cytosol in both treatments compared with the negative controls (Figure 1e). Moreover, we analyzed the co-localization using Pearson correlation coefficient. Cells treated with P2Et fraction or Dx had lower Pearson correlation coefficients than untreated cells, indicating a reduction of cytochrome c in the mitochondria following P2Et exposure (Figure 1f). Altogether these results suggest that the P2Et fraction induces intrinsic apoptotic cell death in B16F10 melanoma cells.

### Apoptosis induced by P2Et treatment is accompanied by the expression of molecular markers of ICD

**Figure 2:**
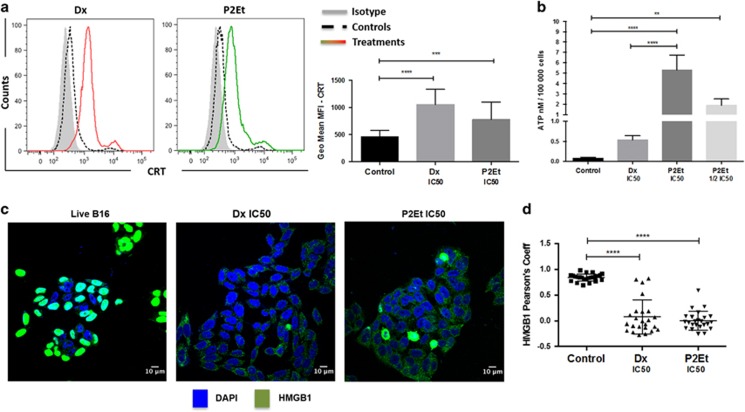
P2Et fraction induces immunogenic B16F10 cell death. B16F10 melanoma cells were cultured in complete media and treated with P2Et IC50 (63.5 *μ*l/ml), Dx IC50 (0.06 *μ*g/ml) or with the negatives controls (EtOH and DMSO—vehicles), for 24 h in all cases. (**a**) CRT: the surface exposure of CRT was determined by flow cytometry among viable cells (Aqua vivid negative), comparisons between negative controls (dashed line—black), positive control Dx (red line) and P2Et-treated cells (green line) and a isotype control (solid histogram). Bars represent the geometric mean values±S.E.M. (*n*=5), ***P<0.001; ****P<0.0001. (**b**) ATP: cells were maintained in control conditions or treated with P2Et IC50, Dx IC50, followed by the assessment of ATP secretion in culture supernatants by luminescence. Quantitative data are means±S.E.M. (*n*=3). **P<0.01; ***P<0.001; ****P<0.0001. (**c**) HMGB1: mobilization was determined by confocal microscopy. Images were acquired with Olympus FV1000 with a 60 × PlanAPO objective and are representative of three independent experiments. Primary antibody for HMGB1 (rabbit anti-mouse) was detected using conjugate goat anti-rabbit secondary antibody (Alexa Fluor 488—green) and nuclei stained with DAPI. (**d**) Pearson correlation coefficients: the distribution coefficients for HMGB1, each dot is one cell for a total of 50 cells per group, values are means±S.E.M. (*n*=3) **** *P*<0.0001

Immune system activation by ICD relies on the production of several danger-associated molecular patterns (DAMPs) such as ecto-CRT at the plasma membrane or secreted CRT, HMGB1 and ATP. To assess whether tumor cell death induced by exposure to P2Et fraction could lead to ICD, we monitored ecto-CRT appearance by flow cytometry on treated B16F10 for 24 h. The geometric mean of the fluorescence intensity (geometrical mean MFI) signal of P2Et and Dx-treated cells was significantly higher compared with non-treated cells (Figure 2a). Similarly, extracellular ATP levels after P2Et or Dx treatment was higher and dose dependent, compared with control conditions (Figure 2b). Finally, HMGB1 mobilisation was determined after 24 h of treatment by confocal microscopy. In control conditions, HMGB1 protein (green) was localized in the nuclei (blue), while the protein was mobilized to the cytoplasm in the Dx and P2Et-treated cells (Figure 2c). The Pearson correlation coefficient was lower in treated cells, indicating that HMGB1 was mobilized from the nuclei to the cytoplasm (Figure 2d), which can be followed by extracellular release.^27^ Altogether, these results indicate that P2Et induces the hallmarks of ICD in B16F10 melanoma cells.

### Apoptosis induced by P2Et treatment is accompanied by autophagy induction

**Figure 3:**
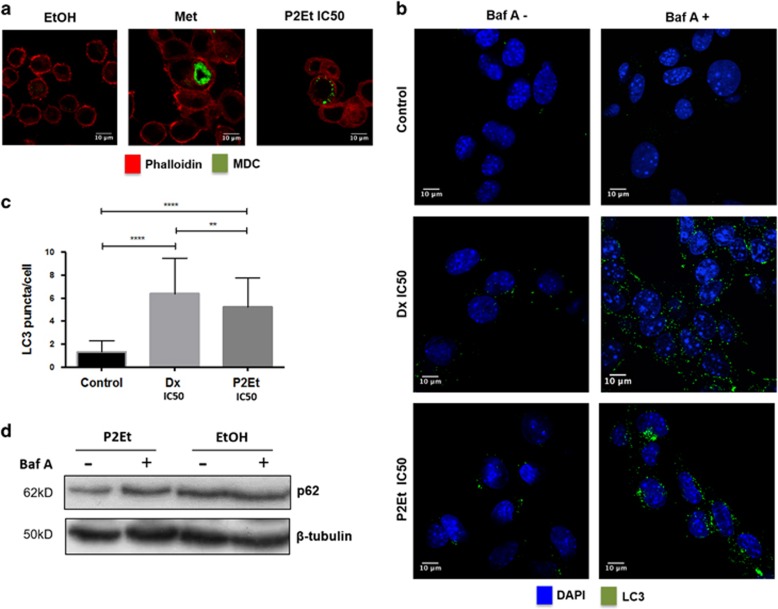
P2Et treatment induces autophagy in B16F10 melanoma cells. Cells were cultures in complete media for 48 h and then treated with P2Et 1/2 IC50, Dx IC50, Met 500 μM or with the negative controls (EtOH and DMSO, vehicles). Autophagy induction was evaluated by two different methods. Images were acquired in a Olympus FV1000 microscopy with a 60 × PlanAPO objective. (**a**) Cells were cultured for 24 h with P2Et, Met (autophagy inducer, positive control), Dx or negative control, then stained with 0.05 mM MDC (green) in complete media for 1 h a 37 °C washed and actin filaments stained with phalloidin Alexa Fluor 594 (red) (*n*=3). (**b**) Cells were cultured for 12 h with P2Et, Dx (autophagy and ICD inducer, positive control) or in control conditions. The last 3 h of the culture (10 nM) bafilomycn A was added to the media as an inhibitor of autophagic flux. Cells were stained the rabbit anti-mouse LC3 polyclonal primary antibody and reveled with a rabbit polyclonal Alexa Fluor 488 secondary antibody (green). DAPI was used to stain the nuclei (blue). (**c**) Quantitative data (Mean±S.E.M., *n*=2) is reported ****P<0,0001; **P<0,01 (*n*=3). (**d**) Cells were treated with P2Et or with the negative control (EtOH) for 24 h. Then, lysates were evaluated for the presence of p62 protein by western-blot

Autophagy has been described as a double-edged sword,^28, 29^ However, autophagy seems to be required for ICD through ATP secretion.^9, 30^ We set out to evaluate autophagy induction by P2Et treatment to further characterize the induction of ICD. B16F10 cells were cultured in the presence of metformine, P2Et or Dx and autophagy induction was determined by two different staining methods using confocal microscopy. First, B16F10 cells treated for 24 h were stained with monodansulcadaverine (MDC) dye (green) followed by actin filaments staining with phalloidin (red). Autophagosome formation was clearly detectable (green dots) on cells treated either with P2Et (IC50 or 1/2 IC50) or with Dx but not in the control conditions (Figure 3a). To confirm these results, we evaluated the presence of the autophagy specific protein LC3 after 12 h with P2Et, Dx or vehicle. When needed, bafilomycin A1 was added 3 h before the cell staining, to inhibit autophagic flux. Autophagy was indeed higher in treated cells as seen by increased LC3 puncta per cell comparing to controls (Figures 3b and c) (mean±S.D., *n*=3). These data were also confirmed by western blot showing higher nucleoporin p62 (p62) degradation, after P2Et and Dx treatments compared with the controls (Figure 3d). These results suggest that the P2Et-induced ICD phenotype is accompanied by the concomitant induction of autophagy.

### P2Et and Dx treatments induce ecto-CRT associated to vesicles at the surface of autophagic B16F10 melanoma cells

**Figure 4:**
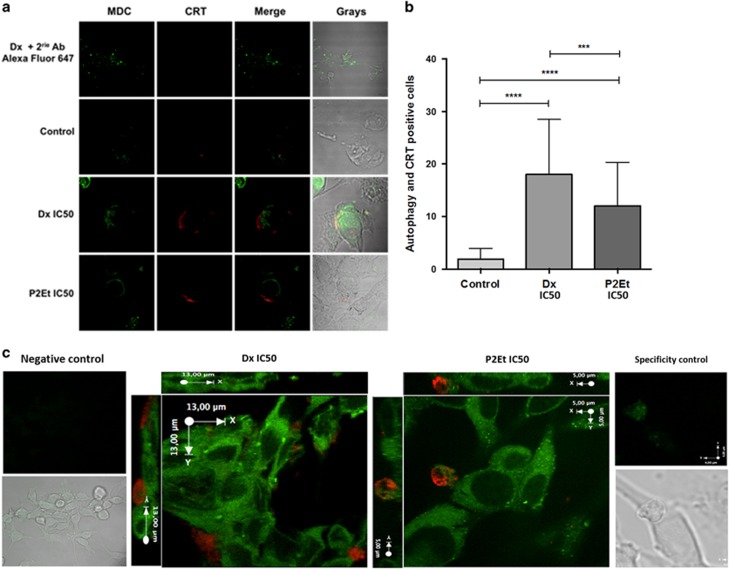
P2Et treatment induces CRT vesicles in the surface of autophagic B16F10 cells. Cells were cultures in complete media and then treated for 24 h with P2Et IC50, Dx IC50 or with the negative controls (Etoh and DMSO). MDC staining was made as described in Figure 3 (green) and CRT was detected with the rabbit polyclonal anti-CRT primary antibody followed by the Alexa Fluor 647 secondary antibody (red). Images were acquired in a Olympus FV1000 microscopy with a 60 × PlanAPO objective. (**a**) Additional control was made with Dx-treated cells stained only with the secondary antibody and MDC (*n*=3). (**b**) The number of CRT and autophagy-positive cells were determined for each treatment counting 50 cells per treatment in different fields. Values are means±S.E.M. ***P<0.001;****P<0.0001 (*n*=3). (**c**) × 2 Zoom image corresponding to B16F10 cells treated with P2Et IC50. *XYZ* plane from an acquisition as followed, *X* (0.33 *μ*m), *Y* (0.33 *μ*m) and *Z* (0.2 *μ*m interval)(*n*=3)

In addition to the expression of individual DAMPs, we investigated whether single cells could express more than one signal at the same time. Therefore we evaluated autophagy induction along with ecto-CRT expression. B16F10 cells were treated for 24 h with P2Et, Dx, or negative controls and autophagy was determined by MDC positive autophagosomes detection (green) and CRT expression (red). We observed significantly higher numbers of autophagy and CRT double positive cells after P2Et or Dx treatment compared with controls (Figures 4a and b), in agreement with the results described in Figures 2 and 3. Interestingly, we also found that both treatments induced vesicles exposing CRT on the surface of autophagic cells (Figure 4c).

### Vaccination with P2Et pre-treated B16F10 cells delays tumor growth

**Figure 5:**
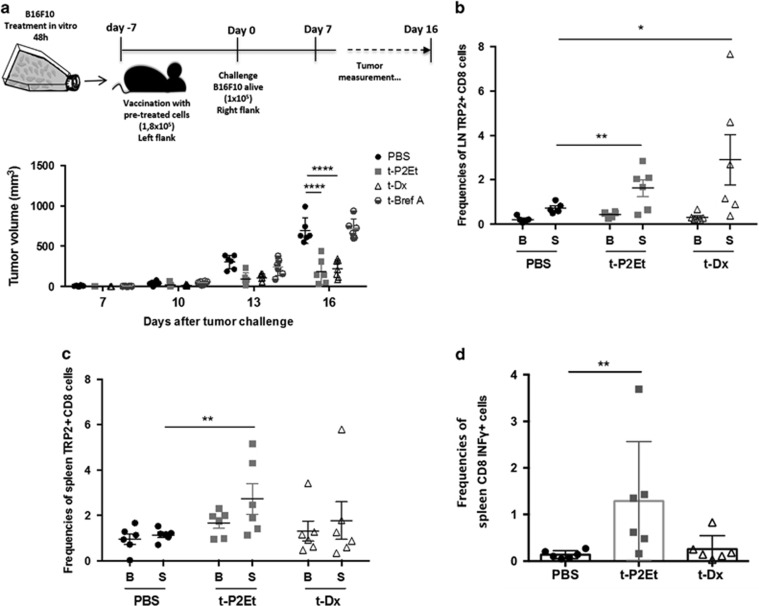
Immunogenicity of different cell death types and antigen-specific response. (**a**) B16F10 cells were treated for 48 h with 101.6 *μ*g/ml P2Et, 0.2 *μ*g/ml Dx (ICD inducer) or for 24 h with 4* μ*l of BrefA (no ICD inducer) to obtain between 60 and 75% of cell death. 1.8 × 10^5^ pre-treated cells (P2Et, Dx and BrefA) were injected s.c. in the right flank; 7 days after mice were challenged with 1 × 10^5^ B16F10 cells alive in the left flank. We include an additional group that did not received vaccination but only alive B16F10 cells as tumor growth control for a total of 4 groups of 8 mice each *n*=2. Tumor evolution was monitored every 2 days. Each dot represents one indivudual. (**b** and **c**) Lymph nodes and spleens cells of vaccinated mice were harvested and expanded *in vitro with* IL2 and IL7 for 8 days and stimulated with Trp2 peptide (S) or left in basal conditions without peptide (B). After expansion antigen-specific cells were detected by tetramer staining. (**d**) Spleen expanded cells were re-stimulated for 6 h for intracellular cytokine staining. In all cases mean±S.D. are represented and *n*=2. *P<0,05; **P<0,01, ****P<0,0001

Anthracycline-treated tumor cells are effective in eliciting anti-tumor IR by ICD whereas other cytotoxic agents, such as Brefeldin A, do not induce ICD nor tumor control. We evaluated whether P2Et pre-treated cells showing molecular markers of ICD could also enhance anti-tumor IR in our *in vivo* model. Thus, we exposed B16F10 cells to Dx, Brefeldin A or P2Et fraction for 48 h *in vitro* and verified apoptosis induction (Supplementary Figure S1A). Immunocompetent C57BL/6 mice were vaccinated with normalized numbers of dying cells in the right flank, which in some cases generated small tumors that did not grow over time, and therefore were not monitored. Instead, mice were challenged 7 days later with live B16F10 tumor cells into the left flank. Protection or delay in tumor growth was interpreted as a sign of effective anti-tumor vaccination. B16F10 pre-treated with P2Et (t-P2Et) fraction were able to induce retardation of tumor growth compared with controls (mice without vaccination but injected with live B16F10) or B16F10 brefeldin A (BrefA) pre-treated group. Dx pre-treated cells (t-Dx) also induced protection as expected (Figure 5a). In addition we observed that t-P2Et mice had higher frequencies of activated (CD44^+^) and central memory (CD62L^+^, CD44^+^) CD8 T cells compare with t-Dx vaccinated or unvaccinated mice in the spleen (Supplementary Figures S1B and C).

As vaccination is considered the functional correlate of cell death immunogenicity, our results suggest that P2Et-mediated cell death of B16F10 cells was indeed immunogenic. To test this hypothesis, we assessed the induction of an antigen-specific IR against the well-known melanoma antigen tyrosine-related protein 2 (Trp2). Lymph node and spleen cells from vaccinated animals were harvested and after 8 days of *in vitro* expansion, Trp2 tetramer staining revealed increased frequencies of antigen-specific cells in the lymph nodes of the mice that were vaccinated with t-P2Et or t-Dx compared to the non-vaccinated ones (Figure 5b). On the other hand, tetramer staining in the spleen showed increase of Trp2-specific CD8 T-cell frequencies only when vaccinated with t-P2Et (Figure 5c). Furthermore, the analysis of intracellular cytokines produced by CD8^+^ T lymphocytes in the spleen revealed an increase in the frequency of INF-*γ* positive cells in the t-P2Et vaccinated mice compared to t-Dx and non-vaccinated animals (Figure 5d).

### Subcutaneous P2Et treatment delays melanoma tumor growth in an immune system-dependent manner partially dependent on T cells

**Figure 6:**
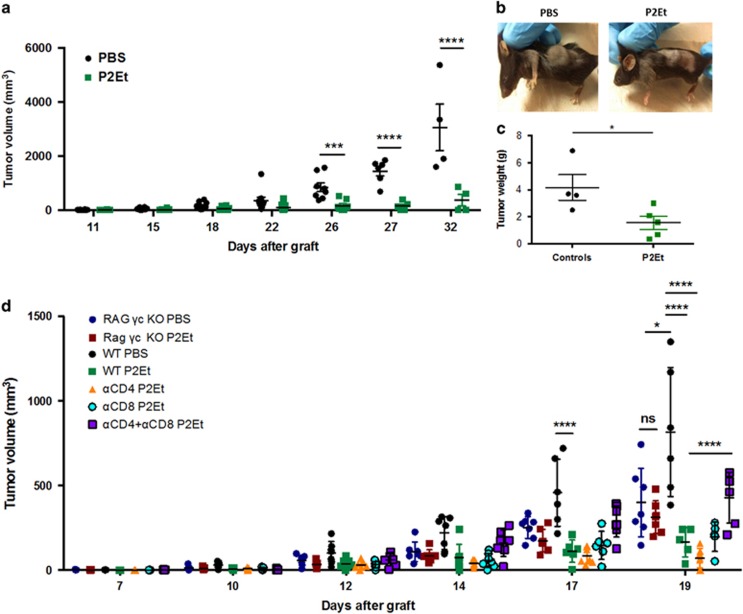
P2Et *in vivo* anti-tumor activity is partially dependent on T cells. Two groups of 10 animals each were injected with 1 × 10^5^ B16F10 cells subcutaneously. P2Et treatment (75 mg/kg) started at day 2 post-engraftment and were made three times a week. The control group only received PBS injections. (**a**) Tumor volume was monitored. Each dot represents one individual. (**b**) Representative images of mice in each group. (**c**) Tumor weight was evaluated at sacrifice. mean±S.D. is represented *P<0,05; ***P<0,001; ****P<0,0001. *n*=5. (**d**) Two groups of C57BL/6 (WT), two groups of Rag *γ*c double KO, one group with depleted CD4, one group of depleted CD8 and one group of co-depleted CD4 and CD8, with 7 animals each, were injected with 1 × 10^5^ B16F10 cells subcutaneously. P2Et treatment (75 mg/kg) started at day 3 post-engraftment and was repeated twice a week. The control groups WT and Rag *γ*c double KO only received PBS injections. I.p. antibody injections for single and double depletions started two days before tumor graft and were repeated every 5 days until the end of the experiment. Tumor volume was monitored. Each dot represents one individual. Mean±S.D. is represented and *n*=2. ***P<0,001; ****P<0,0001

In order to determine if *in vivo* treatment could directly have an anti-tumor effect, two groups of C57BL/6 mice, were engrafted with B16F10 melanoma cells. Two days after tumor engraftment, one group received s.c. P2Et treatment (75 mg/kg) three times per week whereas the second group received phosphate-buffered saline (PBS). P2Et treatment delayed tumor growth compared with control group and differences were significant from day 26 onwards (Figures 6a and b). Tumor weight was also lower in treated mice compared with controls (Figure 6c; *P>0,05; ****P>0,0001; two-way ANOVA). Taken together, these results confirmed that P2Et fraction has anti-tumor activity *in vivo* in the B16 melanoma model.

To assess whether the anti-tumor activity of P2Et required the participation of the host's immune system, we compared the *in vivo* P2Et activity in tumor-bearing immunodeficient mice and wild-type (WT) mice. Briefly, one group of WT C57BL/6 and one group of Rag common *γ* chain knockout (KO) mice were treated s.c. with P2Et two times per week whereas control groups (WT and Rag *γ*c KO) received only PBS at the same time points. We observed that *in vivo* P2Et treatment significantly delayed tumor growth in immunocompetent mice but not in animals lacking T lymphocytes and natural killer (NK) cells, suggesting that P2Et-mediated anti-tumor activity required an intact immune system. To further dissect the role of T cells in P2Et anti-tumor activity, CD4 and/or CD8 antibody-mediated depletions were performed in B16 tumor-bearing mice treated with P2Et as described (Supplementary Fig S2). Strikingly, single depletions of CD4 or CD8 T cells did not significantly affect the P2Et anti-tumor effect. However, the effect of *in vivo* P2Et treatment was partially lost when simultaneous depletion of CD4 and CD8 T cells was done, suggesting that both CD4 and CD8 T cells were important for P2Et anti-tumor activity (Figure 6d).

Finally, to evaluate the role of the innate IR in P2Et-mediated anti-tumor activity, we performed NK cell depletion, and also tested the P2Et fraction in CD1d KO mice to evaluate a possible role of type 1 and type 2 natural killer T (NKT) cells. However, we did not observe any loss of P2Et activity in these two settings, suggesting that NK cells and CD1d-restricted NKT cells are not necessary for P2Et treatment to be effective (data not shown).

### P2Et induces DC activation and increases the *in vivo* antigenicity of B16OVA tumor cells

**Figure 7:**
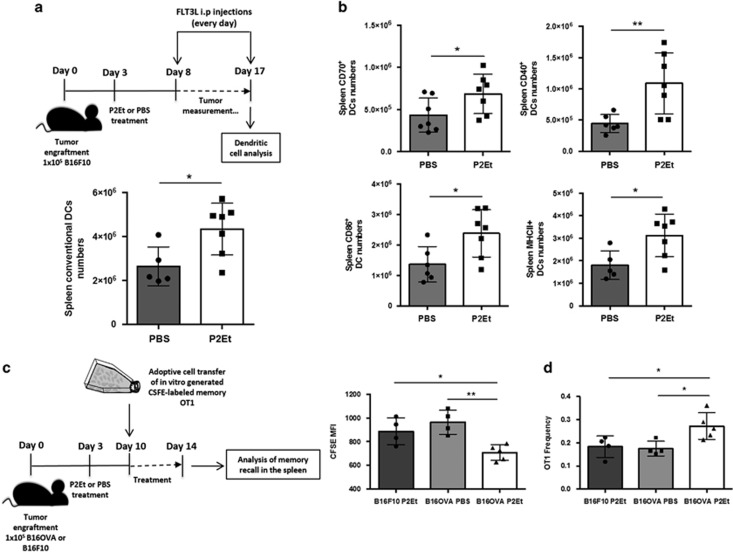
P2Et *in vivo* treatment enhances tumor immunogenicity and DC activation. Six C57Bl/6 mice per group were engrafted with 1 × 10^5^ B16F10 s.c. cells and treated s.c. with P2Et (75 mg/ml) or PBS twice a week until the end of the experiment. At day 8 post-engraftment i.p. FTL3 ligand treatment started and continued every day also until the end of the experiment. At day 17 after tumor engraftment mice were sacrificed, LN and spleens were collected and DCs numbers and phenotypes were analyzed by flow cytometry. (**a**) Upper panel shows the experimental protocol. Lower panel depicts the numbers of spleen conventional DCs (CD45^+^, CD220^-^ and CD11c^+^). (**b**) Histograms represents the number of conventional DCs expressing co-stimulatory molecules. (**c**) Five C57BL/6 mice per group were engrafted with 1 × 10^5^ B16OVA or B16F10 cells s.c. and treated with P2Et as in **a**. At day 10 after engraftment, mice were adoptively transferred with 1 × 10^6^ CFSE-labeled memory OT1 cells and killed 4 days later. (**d**) Left panel, spleens were collected and the MFI of CFSE memory OT1 T cells were analyzed. Right panel, the graph illustrates the frequencies of memory OT1 cells in the spleen. In all cases, each dot corresponds to one individual. Mean±S.D. is represented **P<0.01; *P<0.05

To induce an adaptive IR by ICD or any other type of cell death, the response must go through DC maturation and activation. Accordingly, we decided to assess DC activation *in vivo* after P2Et treatment. Briefly, C57BL/6 mice were engrafted with B16F10 melanoma cells and treated s.c. with P2Et or PBS twice per week. In addition, both groups were treated with FLT3 ligand to increase DC numbers and allow a better resolution. FTL3 ligand treatment did not interfere with P2Et anti-tumor activity, which was highly significant (Supplementary Figure S3A), and the gating strategy for DC analysis is in Supplementary Figure S3B. We observed, that P2Et-treated mice had higher numbers of spleen conventional DCs (CD45^+^, CD220^−^, CD11c^+^) with increased surface expression of co-stimulatory molecules such as CD86, CD40, MHCII and CD70, suggesting that *in vivo* P2Et treatment actually enhances the immunogenicity of tumor cells (Figures 7a and b). In parallel, we performed *in vitro* co-culture of P2Et- or Dx-treated carboxyfluorescein succinimidyl ester (CFSE)-labeled B16F10 cells with CD11b^+^ bone marrow dendritic cells (BMDCs) to evaluate phagocytosis. We observed that 12 h after P2Et or Dx treatment, phagocytosis of B16F10 cells by BMDCs was increased compared with the negative controls (Supplementary Figure S4). Furthermore, P2Et treatment induced higher percentages of CFSE^+^ and CD11b^+^ double positive cells than Dx treatment, (Supplementary Figure S4) suggesting that P2Et fraction enhances a type of cell death, which favors phagocytosis compared with Dx or negative controls. In addition, as P2Et and Dx induce CRT externalization, which is seen as an ‘eat-me' signal, we also analyzed by confocal microscopy the co-culture of BMDCs with CFSE-labeled B16F10 treated with P2Et or Dx. We observed that BMDCs efficiently phagocytosed P2Et or Dx-treated B16F10 cells. In addition, CRT expression was specifically observed in P2Et-treated cells, as compared with solvent (Supplementary Figure S5). Interestingly, the interaction between B16F10 cells and BMDCs was seen in high CRT concentration zones. However, we also found CRT on the surface of BMDCs, which prevented us, to determine if the CRT only originated from B16F10 or also from BMDCs.

To further verify the hypothesis about immunogenicity enhancement, we used the B16OVA model as a tool to evaluate the memory recall after P2Et treatment. To address this question, we engrafted C57BL/6 mice with B16F10 or B16OVA tumor cells and treated the mice with P2Et or PBS using the same protocol mentioned before (Figure 7c). Memory OT1 cells were generated *in vitro* as described in the methods, and 1x10^6^ CFSE-labeled memory OT1 cells were adoptively transferred at day 10 post-tumor engraftment. After sacrifice 4 days later, we observed that spleen OT1 cells showed a lower CFSE-MFI only in mice with B16OVA treated with P2Et, suggesting an antigen-specific increased T-cell proliferation (Figure 7d, left panel). Indeed, higher frequencies of OT-1T cells were detected only in B16OVA/P2Et-treated mice as compared to PBS or B16F10/P2Et groups (Figure 7d, right panel). These results suggest that after P2Et treatment more antigen might be available to induce a faster memory recall, thus OT1 cells proliferation. All together, these results re-enforce the hypothesis that P2Et treatment enhances tumor cells immunogenicity and that membrane-exposed CRT might interact with DCs to participate in their activation and the enhancement of tumor antigen phagocytosis.

## Discussion

The main findings reported in this study are (i) that a well-characterized plant extract causes immunogenic cell death in melanoma tumor cells *in vitro* and (ii) that the induced anti-tumor immunity *in vivo*, either through vaccination with dying tumor cells or as a result of direct treatment with the extract, mediates significant disease control associated with a detectable tumor-specific CD8 T-cell response.

Plants are considered among the main sources of biologically active products with an estimated 50% of the prescription products available in Europe and the USA.^31^ In spite of the recent domination of the synthetic chemistry as a method to discover and produce drugs, the potential of entire plants or their bioactive fractions remains attractive to provide new therapies. In particular, plant-derived drugs such as vinblastine, vincristine, taxol and camptothecin have significantly contributed to reinforce the anti-tumor chemotherapy arsenal.^32^ Until now, these therapies have been useful despite the emergence of resistant clones over time that contributes to the development of late metastasis and decrease disease-free survival.^33, 34^ Current knowledge of cancer complexity as well as the close dependence on the microenvironment, has allowed us to understand that combinatorial anti-tumor therapies hold great promise in cancer patients,^35, 36^ especially when therapy is directed against tumor cells and induces the activation of an IR that later can actively participate in the destruction of metastatic cells.^37^

Complex fractions obtained from plants constitute a natural source of compounds that act in synergy enhancing their biological activity especially when plants have a history of traditional use in the treatment of cancer. We previously described the cytotoxic activity of P2Et and that the fraction is not cytotoxic on normal activated PMBCs, or normal fibroblast, suggesting a certain selectivity of the treatment and potentially resulting in fewer side effects. Anti-tumor activity of polyphenols includes the induction of tumor cell apoptosis^25^ and inhibition of drug resistance pumps^38^ among others, which may also increase the sensitivity of tumor cells to other drugs.^39, 40^ Indeed, one advantage of the plants fractions transformed into phytomedicines, is their *in vivo* low toxicity due to the relative low concentration of molecules with cytotoxic activity.^41, 42, 43^

Recently, we showed that P2Et can also induce some of the features of ICD *in vitro*, together with anti-tumor and anti-metastatic effects in the mouse breast carcinoma model 4T1.^20^ However, this model did not allow us to determine the generation of tumor antigen-specific T cells. In the present study, we show that treatment with a standardized fraction obtained from a plant, confers protection against tumor growth in the B16 mouse melanoma model through DCs activation and the generation of an adaptive antigen-specific IR that increases the frequencies of CD8^+^ IFN-*γ*^+^ T cells.

The concept of ICD,^2, 3^ where dying cells are converted into an *in situ* vaccine^44^ induced by some type of chemotherapeutic drugs such as anthracyclines among a few others drugs, is characterized by the generation of ‘danger signals' or DAMPs. These signals make tumor cells ‘visible' to the immune effector cells and induce an IR against antigens from self dying cells while braking tolerance. Factors contributing to ICD involve changes in the composition of the cell membrane as well as the release of soluble mediators. We show that *in vitro* P2Et treatment actually induces apoptosis along with all the ICD molecular markers and that during this process, we observed CRT-charged vesicles exposed at the cell surface. Moreover, we showed using the B16OVA model that *in vivo* P2Et treatment might increase the amount of available antigen inducing proliferation of OTI cells. These signals trigger DC activation and maturation *in vitro* and *in vivo*. Fully activated and matured DCs can migrate to secondary lymphoid organs and activate antigen-specific naive T lymphocyte in an efficient manner.^2^ In our hands, melanoma cells treated with various doses of P2Et did promote phagocytosis, probably involving CRT as an ‘eat-me' signal, and cross priming of bone marrow-derived dendritic cells, which was indirectly evidenced by the *in vivo* generation of IFN-*γ* producing, Trp2-specific CD8+T cells.

The functional correlate for the ability of a given compound to trigger ICD is the ability of dying cell to vaccinate when administered into an immunocompetent host.^45^ Vaccination with P2Et pre-treated B16F10 tumor cells did confer significant tumor growth retardation and favored the induction of central memory CD8 T cells following inoculation of viable tumor cells. Moreover, the anti-tumor effect induced by direct treatment with P2Et of tumor-bearing mice was dependent to a large extent on an intact immune system and at least in part on T cells, as demonstrated by the loss of anti-tumor response in Rag^−/−^ *γ*c^−/−^ KO mice with concomitant depletion of CD4 and CD8 T cells. Interestingly, we noted as others that immunodeficient animals developed smaller tumors even in the absence of P2Et treatment. This phenomenon could be due to the inability of these animals to develop an inflammatory response, dependent on the presence of IFN-*γ* and NK cells^46, 47^ as well as on the absence of regulatory T cells known to help tumor growth and vascularization.^48^

Altogether, our data indicate that the P2Et polyphenol rich extract is an ICD inducer in the *in vivo* B16 melanoma model, and may constitute an effective phyto-based agent in the treatment of melanoma and perhaps other tumor types including breast carcinoma. Thus, additional preclinical and clinical studies in therapeutic settings are warranted.

## Materials and Methods

### Reagents

The H-2K^b^-restricted CD8 T-cell epitope trp2_180–188_ (SVYDFFVWL) synthetic peptide was obtained from the University of Lausanne in-house facility. Phycoerythrin-conjugated H-2K^b^/trp2_180–188_ tetramers were purchased from TCMetrix, Lausanne, Switzerland. Human recombinant interleukin 2 (rh-IL2) was obtained from GLAXO IMB; human recombinant interleukin 7 (rh-IL7) from PeproTech (Hamburg, Germany); GolgiStop and Cytofix/Cytoperm from BD Biosciences (Allschwil, Switzerland); PE-conjugated rat anti-mouse IL2 monoclonal antibody from BD Pharmigen (Allschwil, Switzerland); pacific blue-conjugated anti-mouse TNF-α monoclonal antibody from Biolegend (San Diego, CA, USA); FITC-conjugated anti-mouse INF*γ*, Alexa Fluor 700-conjugated anti-mouse CD3, Percpcy5.5-conjugated anti-mouse CD4 and PE-Cy7-conjugated anti-mouse monoclonal antibodies from eBiosciences (Frankfurt, Germany); Rabbit polyclonal anti-CRT primary antibody (2907), rabbit polyclonal anti-HMGB1 (79823) from Abcam (Boston, MA, USA); goat anti-rabbit secondary antibody conjugated with Alexa Fluor 488 flurochrome, mouse anti-rat Alexa Fluor 647, mouse anti-rabbit Alexa Fluor 488 secondary antibody from Molecular probes–Invitrogen; rat anti-mouse anti-cytochrome c monoclonal primary antibody clone 6H2 (13561) from Santa Cruz Biotechnology (Santa Cruz, CA, USA); rabbit polyclonal LC3A/B (4108) from Cell Signaling Technology (Danvers, MA, USA); anti-nucleoporin p62 clone 53 (610498) purified antibody from Becton-Dickinson (San Jose, CA, USA); goat anti-rabbit horseradish peroxidase-conjugated secondary antibody from Thermo Fisher Scientific; DAPI (4′,6-diamidino-2-phenylindole), MDC and propidium iodide (PI) from Sigma-Aldrich (Carlsbad, CA, USA); Mito-Tracker Red FM, Alexa Fluor 594-conjugated phalloidin and Annexin V-Alexa Fluor 488 from Molecular probes- Invitrogen (Chelmsford, MA, USA); CellTrace CSFE from Thermo Fisher Scientific and LIVE/DEAD Aqua from Thermo Fisher Scientific (Chelmsford, MA, USA). APC conjugated anti-CD62L, APC conjugated anti CD11c, APCefluor780 conjugated anti-CD45.2, PE-Cy7-conjugated anti-CD45.1, PercpCy5.5-conjugated anti-CD70, PE-conjugated anti-CD40, conjugated anti-CD44 monoclonal antibodies from eBiosciences. PE-Texas Red conjugated anti-B220, Pacific blue-conjugated anti-CD11b, FITC conjugated and PercpCy5.5 anti-MHCII monoclonal antibodies from BD. Anti-CD4 (GK.1.5) from LICR FACs facility and anti-CD8 (53-6.72) from BioXcell (Lebanon, NH, USA) depleting antibodies.

### Mice

C57BL/6 mice were purchased from Charles River (Wilmington, MA, USA) and maintained at the Pontificia Universidad Javeriana following the established protocols of the Ethics Committee of the Sciences Faculty and National and International Legislation for Live Animal Experimentation (Colombia Republic, Resolution 08430, 1993). Immunodeficient mice recombination-activating genes (RAG)2x common cytokine receptor gamma chain double mutants have been previously described^49^ and were maintained at the University of Lausanne's specific pathogen free facility in accordance with Swiss ethical guidelines. Age and sex-matched mice between 5 and 10 weeks of age were used for all experiments. The present study was approved by the ethics committee of the Science Faculty at a meeting on 6 May 2012.

### Cell lines and culture conditions

Melanoma B16F10 and B16OVA cell lines were kindly provided by PR (Ludwig Center for Cancer Research, Department of Oncology—Faculty of Biology and Medicine University of Lausanne, Switzerland). Cells were cultured in RPMI-1640 (Eurobio, Toulouse, France) supplemented with heat-inactivated fetal calf serum (10%) (Eurobio), 2 mM  l-glutamine, 100 U/ml penicillin, 100 *μ*g/ml streptomycin, 0.01 M Hepes buffer and 1 mM sodium pyruvate (Eurobio) and incubated in a humidified environment at 37 °C and 5% CO_2_. Cells were grown until 75% confluence and passaged using trypsin/EDTA (ethylene-diamine-tetra-acetic acid) 1 × (Eurobio), washed with PBS and resuspended in supplemented RPMI-1640. Tumor cells were proven Mycoplasma-free using a MycoProbe Mycoplasma Detection Kit (R&D Systems, Minneapolis, MN, USA).

### Mouse BMDC

Briefly, bone marrow cells (BMC) were collected from the femurs of C57BL/6 mice using culture medium (RPMI-1640 supplemented as described before). Following centrifugation, the BMC were cultured in RPMI medium supplemented with 20 ng/ml granulocyte–macrophage colony-stimulating factor (GMCSF; R&D Systems) and 50 *μ*M 2-mercaptoethanol in a humidified 5% CO_2_ incubator at 37 °C. At day 3, half of the culture medium was replaced with fresh medium containing GMCSF and 2-mercaptoethanol. At day 6, the non-adherent and loosely adherent DCs were collected. The DCs produced in this manner were immature BMDCs and displayed typical morphologic features of DCs.

### *In vitro* T-cell expansion and re-stimulation

At the end of the experiment, day 16 after tumor engraftment, spleen and tumor-draining lymph node cells were collected and plated into 24-well plate at a density of 1 × 10^6^ cells per well. Cells were incubated with 50 U/ml of rh-IL2 with or without 1 *μ*g/ml trp2_180–188_ peptide for 4 days. Then media was changed and fresh peptide and rh-IL2 were added for 2 extra days. Finally cells were cultured for the last 2 days with 50 U/ml of rh-IL2 and with 10 ng/ml of rh-IL7.

### Intracellular cytokine staining

After 8 days of *in vitro* re-stimulation, cells were harvested and plated into 96-well plates. Cells were stimulated or not with 5 *μ*g/ml of trp2_180–188_ peptide for 6 h. GolgiStop was added during the last 5 h of re-stimulation. Stimulation with PMA/ionomicin was used as a positive control. Then cells were stained with LIVE/DEAD Aqua followed by surface labeling with anti-CD3, anti-CD4 and anti-CD8 antibodies. Cells were fixed and permeabilized using the Cytofix/Cytoperm kit according to the manufacturer's specifications and intracellular cytokines were detected with anti-IFN-*γ*, anti-TNF-α and anti-IL-2 antibodies.

### *In vitro* cytotoxicity analysis and cell death assay

P2Et fraction and doxorubicin effects on tumor cells were evaluated using methylthiazol tetrazolium assay (MTT) or neutral red assay (NR). In both cases, 4 × 10^3^ cells were seed in 96-well plates. Treatments were added as serial dilutions starting at 250 *μ*g/ml and as far as 0.97 *μ*g/ml 24 h after seeding and leave during 48 h. When treatment was finish media was replaced by 100 *μ*l of new media with out phenol red plus 50 *μ*l of MTT 1x solution or neutral red (40 mg/ml) and incubated for 4 h at 37 °C. For MTT the crystals were dissolved with 100 *μ*l of DMSO and for neutral red the same volume of a discoloration (EtOH 50%, H_2_O 49% and glacial acetic acid 1%) solution was used. In both cases DO was measured at 540 nm after 20 min after of incubation in a Multiskan flow cytometry (FC; Thermo Scientific, Waltham, USA).

The IC50 (50% inhibition of cell growth) value was calculated using a no linear regression log (inhibitor) *versus* response–variable slope graph in GraphPad Prism (GraphPad Prism Software, La Jolla, CA USA). Cell death was quantified using Annexin V and PI, as previously reported.^25^ Duplicate samples were acquired on a FACSAriaII (Becton-Dickinson) and analyzed with FlowJo software (Tree Star, Ashland OR, USA).

### Caspase activity assays

Caspase 3 activity was assessed using a caspase 3 colorimetric assay kit (Sigma-Aldrich), whereas caspase 9 activity was evaluated using ApoTarget caspase 9 Protease Assay (Invitrogen). Briefly, 9 × 10^6^ B16F10 cells were treated with P2Et IC50 and 1/2 IC50, doxorubicin IC50, curcumin IC50 (positive controls) or ethanol (negative control, 0.02%) for 6, 12 and 24 h for caspase 9 and 48 h for caspase 3. Caspase 3 and 9 activities were estimated following the manufacturer's instructions. The increase in caspase 3 activity was calculated from a calibration curve prepared with p-nitroanilide (pNA) standards using the following formula: Activity, μmol pNA/min/ml=((μmol pNA × *d*)/(*t* × *v*)), where *d*=dilution factor, *t*=reaction time in min and *v*=volume of sample in milliliters. Comparison of the absorbance of pNA from treated samples with an untreated control allows determination of the fold increase in caspase 9 activity.

### Immunofluorescence staining

For all immunofluorescence assays 10^4^ B16F10 cells were seeded onto glass coverslips in 12-well plates and were grown overnight before treatment. Cells were fixed in 4% formaldehyde for 20 min, except for CRT staining were 0.25% formaldehyde for 5 min was used. This was followed by washing (x3) with PBS and permeabilization with Triton X-100 0.1% for 5 min. Blockage was made with PBS 10% FCS for 1 h. Cells were treated for 24 h with P2Et or Dx IC50 (63.5 and 0.06 *μ*g/ml, respectively) or half of IC50. For CRT staining cells were incubated 30 min with anti-CRT primary antibody at room temperature (RT) followed by a 30 min incubation with the Alexa Fluor 488 secondary antibody. The number of autophagy and CRT double positive cells was determined by counting 50 cells per treatment in different fields. HMGB1 staining was made first with the primary antibody anti-HMGB1 for 30 min RT, followed by incubation with the secondary antibody conjugated to Alexa Fluor 488 and finally nuclei were counter stained with DAPI (300 nM) for 20 min. For cytochrome c staining, cells were stained with Mito-Tracker Red FM (250 nM) for 1 h at 37 °C, followed by fixation and permeabilization. Then cells were stained with anti-cytochrome c primary antibody for 30 min followed by secondary antibody Alexa Fluor 647 and DAPI. In the case of autophagy, cells were stained for 1 h with MDC at 37 °C followed by fixation and permeabilization. Actin F staining was made with Alexa Fluor 594 conjugated phalloidin. For LC3 staining, the primary LC3A/B antibody was used overnight followed by detection with Fluor 488 secondary antibody and DAPI. Puncta per cell were counted for 50 cells per treatment in different fields. For autophagy flux studies, bafilomycin A1 was added (1 *μ*M) 3 h before starting the staining. For phagocytosis B16F10 cells were staining with CellTrace CSFE 5 nM for 20 min, grown and treated for 12 h, then washed twice with PBS. 30 × 10^3^ BMDC were added and incubated at 37 °C for 3, 8, 12 and 24 h. Cells were centrifuged, fixed and stained for CRT as described before and stained with anti-CD45.

In all cases coverslips were mounted on slides with ProLong Antifade Reagent (Life Technologies, Woburn, MA, USA) and imaged with a laser scanning confocal microscope FV1000 (Olympus, Conklin, NY, USA) using an UPLSAPO 60 × 1.35 NA oil immersion objective. A 405 nm diode laser was used to visualize DAPI, a 488 nm argon laser line was used for Alexa Fluor 488 nm and a HeNe 543 nm laser was used for Alexa Fluor 594 nm. XY sections at 0.22 *μ*m per pixel and a resolution of 1024 × 1024 or 640 × 640 were obtained. For the Pearson correlation coefficients the analysis was made taking 50 cells per treatment from different fields.

### Flow cytometry

To evaluate cell membrane CRT expression, a total of 9 × 10^5^ cells were plated in 6-well plates and treated the following day with P2Et, DX (positive control) at the IC50 concentration or ethanol (0.05%) for 24 h. Cells were stained as previously described.^20^ Briefly, cells were fixed with 0.25% formaldehyde for 5 min, washed and then stained with the primary anti-CRT antibody followed by the Alexa Fluor 488 secondary antibody and incubated for 30 min. The MFI of stained cells was assessed within the live gate population. For tetramer staining cells were stained first with the MHC-class-I Trp2 tetramer at RT for 30 min, then, surface staining antibodies CD3, CD4 and CD8 were added without wash and incubated for 30 min on ice. For the BMDCs assessment and co-culture the DCs were cultured in complete media and treated with P2Et (72.7 *μ*g/ml), Dx (0.06 *μ*g/ml) or negative controls (ETOH and DMSO) for 36 h at 37 °C. Lipopolysacharide (LPS) stimulation was carried out in parallel as a positive control of BMDCs stimulation (1 *μ*g/ml) during 48 h. BMDCs were stained for 30 min at 4 °C with CD45-PE-Cy5, CD11c-FITC, CD11b-Alexa 700, IAb-BV421, CD40-APC y CD86-PE-Cy7 monoclonal antibodies, (Becton-Dickinson). For Phagocytosis Assay cells were stained with 5 *μ*M CFSE (Life Techonologies), incubated for 20 min at RT, washed twice and plated 2 × 10^5^ cells per well on 6-well plates. Then cells were cultured and treated with P2Et (218.1 μg/ml), using Dx (0.06 *μ*g/ml) as positive control and the negatives controls during 48 h at 37 °C. In addition, B16F10 cells were washed and tripzinized and co-cultured with BMDCs at 1:1 ratio for 12 and 24 h at 37 or 4 °C. Following the interaction, BMDCs were stained with anti-CD45-PE-Cy5, anti-CD86-PE-Cy7 and anti-IAb-BV421. Phagocytosis of apoptotic B16F10 cells by BMDCs was defined by the percentage of double positive cells by fluorescence-activated cell sorting (FACS) analysis. After phagocytosis assay BMDCs activation was assessed, calculating the CD86 geometric mean.

In all cases cells were washed once with PBS 2% FCS and resuspended in 200 *μ*l of PBS 2% for acquisition. Antibodies were diluted in blocking buffer (PBS 2% FBS) and cells were stained with LIVE/DEAD Aqua for death cell exclusion. Samples were acquired using FACSAriaII or LSRII cytometers (Becton-Dickinson) and analyzed with software FlowJo (Tree Star).

### ATP assays

To evaluate ATP release, a total of 9 × 10^4^ cells were treated with P2Et IC50, DX IC50 (positive control), or ethanol for 15 and 24 h, and extracellular ATP was measured by ATP Bioluminescence Assay Kit HS II (Roche, Mannheim, Germany) following the manufacturer's instructions. For assessment of the chemoluminescent signal, the plates were read in a Chameleon V (FI-TRF-FP-Abs-Lum reader) (Hidex).

### Western blotting

Cells were trypsinized, washed twice in PBS and lysed with buffer containing 50 mM Tris-HCL, 150 mM NaCl, (pH 7.5), 1% NP40, 1 mM PMSF, 0.5% sodium deoxycholate and 0.1% SDS. Lysates were centrifuged (14 000 r.p.m. for 8 min at 4 °C) and protein concentration was measured by the Bradford assay (Bio-Rad). Fifteen micrograms of protein were separated by SDS-polyacrylamide gel electrophoresis gels and transferred to PVDF membrane using standard techniques. The membranes were stained with anti-nucleoporin p62. Blots were probed with a 1:5000 dilution of horseradish peroxidase-conjugated secondary antibody. Blots were visualized by using Super Signal West Pico chemiluminescent substrate (Pierce).

### Anti-tumor activity and vaccination

For tumor induction, C57BL/6J mice were shaved on the right flank and engrafted s.c. with 1 × 10^5^ viable B16F10 cells in 100 *μ*l of PBS. For the treatment, P2Et was administrated s.c. around the tumor 3 days after tumor cell challenge. Mice were treated three times per week with 75 mg/kg body weight (bw) (200 *μ*l per mouse). P2Et therapeutic dose was determined as a fourfold lower than the LD-50 (median lethal dose) estimation to ensure low toxicity. Unfortunately, it was not possible to include a group with *in vivo* Dx treatment in these settings, as its bio-availability in the skin has been shown to be low, which did not allow its local application.^50^ To assess the effect of P2Et treatment on IR activation, B16F10 cells (1.8x10^5^)^51^ were treated with 101.5 *μ*g/ml P2Et fraction (t-P2Et), 0.2 *μ*M Doxorubicin (t-Dox) or 4 *μ*l/ml Brefeldine A (t-BrefA) for 48 h. Dying cells were harvested, washed and resuspended in 200 μl of PBS and injected into the right flank of C57BL/6 mice as a vaccine. Seven days later, B16F10 cells (1 × 10^5^) harvested from the exponential phase of growth were suspended in 200 *μ*l of PBS and injected into the left flank (s.c.). In both experimental settings the size of the tumors was assessed three times per week with Vernier calipers and volume was calculated using the following formula: tumor volume (mm^3^)=((width)^2^ × length)/2.

### Depletions

All depletions of CD4, CD8 and NK cells *in vivo* were perform by intraperitoneal (i.p.) injection and started 2 days before tumor engraftment. Depletions of the different populations were verified by blood staining before starting and in two other occasions before the end of the experiment. For CD4 depletion 200 *μ*g per mouse per dose of monoclonal GK1.5 anti-CD4 (ref. 52) were administered the first time but only 100 *μ*g for the following doses. In the case of CD8, mice received 100 *μ*g per mouse per dose of monoclonal 53-6.72 anti-CD8 antibody. In both cases depletions were made every 5 days. Finally, for NK depletion 200ug/mouse/dose of monoclonal anti-NK1.1 antibody was administered every 8 days.

### *Ex vivo* dendritic cell analysis

To be able to analyze dendritic cell population C57BL/6 mice were treated by i.p. injections every day with 20 *μ*l of Fms-like tyrosine kinase 3 ligand (FLT3L) serum that Dr Hans Acha-Orbea kindly provided us. FTL3 ligand serum was obtained from transgenic mice expressing high levels of human FLT3L that display marked expansion of dendritic and myeloid cells leading to splenomegalia and leukocytosis.^53^ Treatment with FLT3L started on day 8 after tumor engrafted and were maintained until the end of the experiment. Spleen, lymph node and tumor cells were collected, stained with LIVE/DEAD Aqua to discard death cells and FC receptors blocked for 20 min before surface staining. With out washing, cells were incubated with the antibody mix two times concentrated for extra 30 min on ice. Markers of DCs phenotype and activation as CD45, CD11c, CD11b, Ly6G, Ly6C, CD8α, B220, CD86, CD40, CD70 and MHCII were assessed.

### OT1 cell transfer

Spleens from OT1 mice described previously^53^ from University of Lausanne facility were harvested and cells were activated and memory cells were generated *in vitro*. 1 × 10^6^ cells were seed in 2 ml in 24-well plates and activated with 1 *μ*M of ovalbumin peptide (SIINFEKL) plus 50 U/ml of rh-IL2. After 2 days media was refreshed and a full dose of IL2 was added. The day after cells were washed and re-plated and rh-IL15 (20 U/ml) was added to the culture media. After 3 extra days, part of the cells were collected to verify their memory phenotype (CD62L^+^ CD44^+^) by FACs. Before transfer cells were labeled with CellTrace CFSE (0.5 *μ*M) and resuspended in PBS. OT1 memory cells (1 × 10^6^) were transfered by intravenous injection and mice were killed 4 days later to established OT1 frequencies and proliferation.

### FLT3 ligand treatment

Serum from FLT3L tg mice described before^53^ was kindly provided by Dr Hans Acha-Orbea from the biochemistry department of the Lausanne University. C57BL/6 mice were engrafted with 1 × 10^5^ B16F10 cells and treated twice a week with 75 mg/kg of P2Et or PBS from day 3 post-engraftment until the end of the experiment. In addition 20 *μ*l/mice of FLT3L serum were brought to a final volume of 100 *μ*l/mice in PBS to be injected i.p. every day starting at day 8 post engraftment until the end of the experiment. This treatment increases the percentage of DCs from 2 to 10% in the spleen.^53^

## Abbreviations

ATP: adenosine triphosphate
BMDC: bone morrow dendritic cells
BrefA: brefeldin A
CFSE: carboxyfluorescein succinimidyl ester
CRT: calreticulin
DAMPs: danger-associated molecular patterns
DAPI: 4′,6-diamidino-2-phenylindole
DC: dendritic cells
Dx: doxorubicin
FACS: fluorescence-activated cell sorting
FC: flow cytometry
FTL3L: fms-like tyrosine kinase-3 ligand
GMCSF: granulocyte–macrophage colony-stimulating factor
HMGB1: high-mobility group box 1
i.p.: intraperitoneal
IC50: half maximal inhibitory concentration
IR: immune response
IFN-*γ*: interferon gamma
KO: knockout
MDC: monodansulcadaverine
MFI: mean fluorescence intensity
MTT: 3-(4,5-dimethylthiazol-2-yl)-2,5-diphenyltetrazolium bromide
NK: natural killer cells
NKT: natural killer T cells
NR: neutral red
OVA: ovalbumin
p62: nucleoporin p62
PBS: phosphate-buffered saline
PI: propidium Iodide
pNA: p-nitroanilide
s.c.: subcutaneous
TCM: central memory T cells
Trp2: tyrosine-related protein 2
WT: wild type
