# Supplementary material for: Immune-system-dependent anti-tumor activity of a plant-derived polyphenol rich fraction in a melanoma mouse model
Source: Cell Death Dis. 2016 Jun 2;7(6):e2243–. doi: 10.1038/cddis.2016.134 (PMC5143373; doi:10.1038/cddis.2016.134)
Supplement: Supplementary Information [file cddis2016134x1.doc]

**Supplementary Information**

**Immune system dependent anti-tumor activity of a plant-derived polyphenol rich fraction in a melanoma mouse model**

**Alejandra Gomez-Cadena^1, 2^, Claudia Urueña^1^, Karol Prieto^1^, Alena Donda^2^, Alfonso Barreto^1^, Pedro Romero^2^, Susana Fiorentino^1^**

**^1^ Grupo de Inmunobiología y Biología Celular. Pontificia Universidad Javeriana, Bogotá - Colombia**

**^2^ Ludwig Cancer Research Center, University of Lausanne, Lausanne – Switzerland**

**Supplementary figure S1**

**
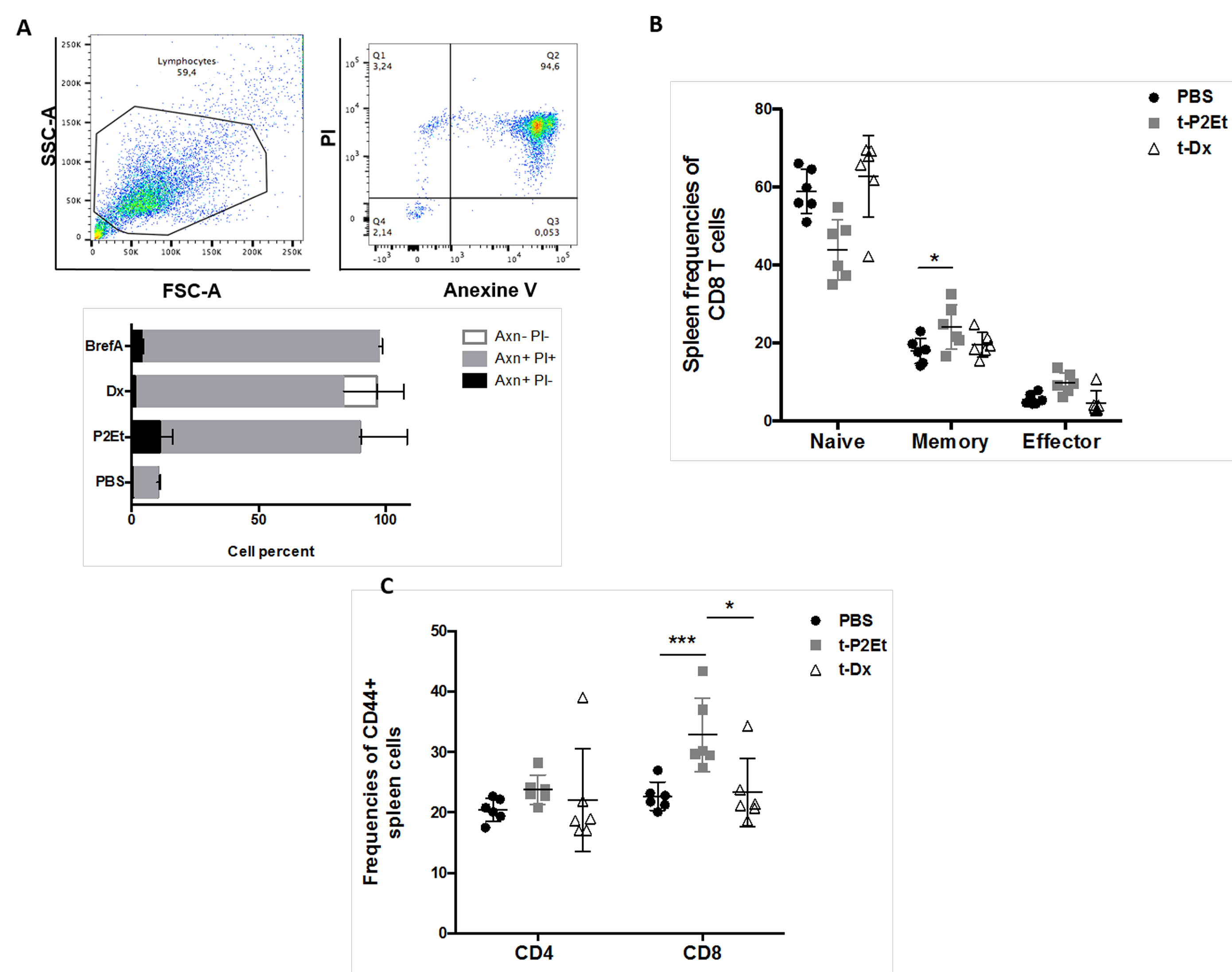
**

**Fig S1. B16F10 pre-treatments**. **A.** 1.8x10^5^ B16F10 melanoma cells were pre-treated in vitro for 48h with P2Et, Dx, Bref A or PBS as a control of viability. Cells were harvested and stained for Annexin V-FITC and PI to determine the percentage of apoptotic cells. Upper panel: Plots of one representative experiment are shown; lower panel: graph represents the respective percentages of live, apoptotic or dead cells; mean±SD is represented. n=2. **B.** Spleens from vaccinated and control mice were harvested and T cell phenotypes were analyzed by flow cytometry. Naïve cells correspond to CD62L^+^CD44^-^, memory to CD62L^+^ CD44^+^ and effectors to CD62L^-^ CD44^+^. n=2. **C.** Activation state of spleen cells of vaccinated mice was evaluated using the CD44 marker. In all cases each dot represents an individual. mean±SD is represented ***P < 0,001; *P < 0,05.

**Supplementary figure S2**

**
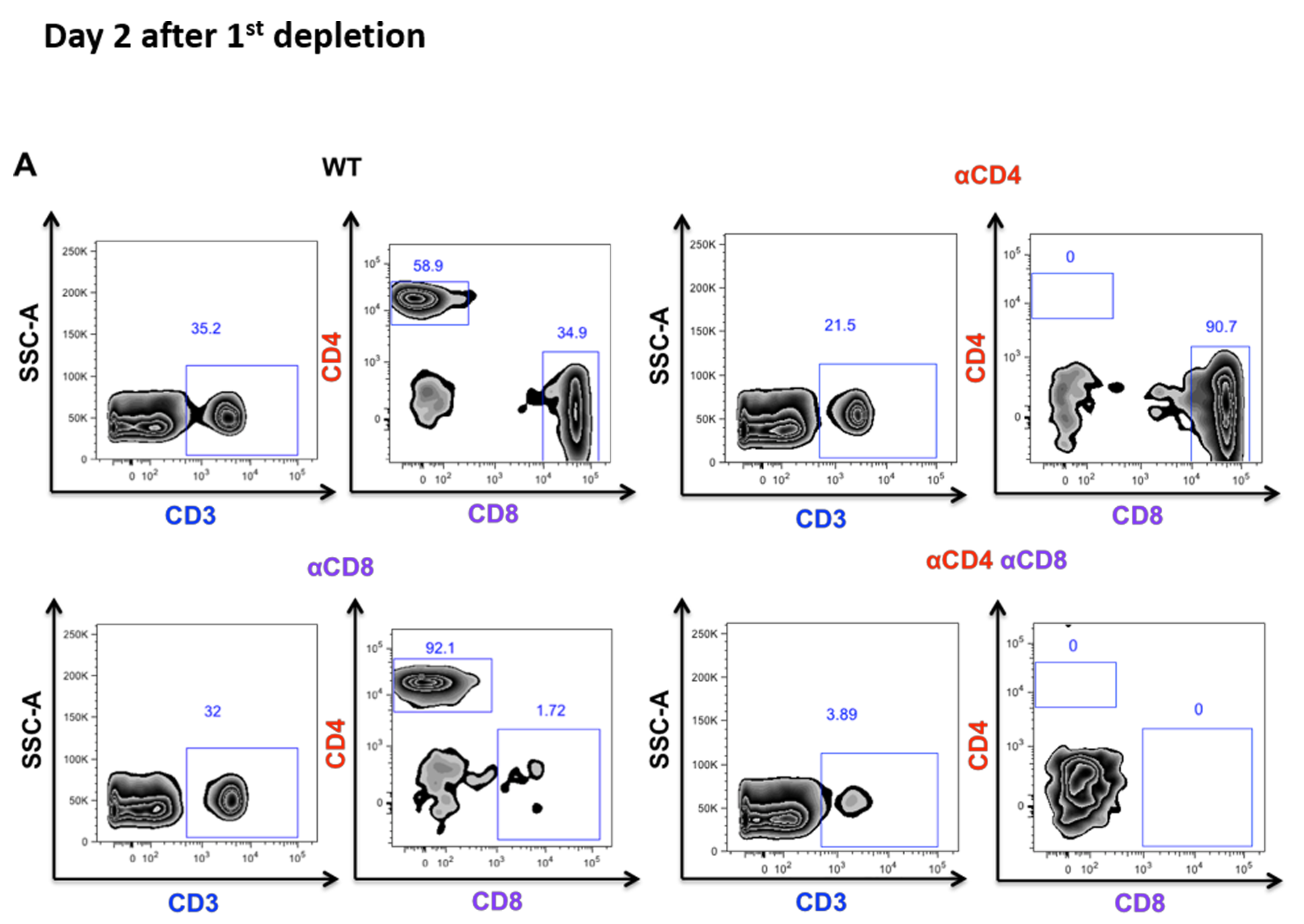
**

**Fig S2. CD4 and CD8 depletions.** Intraperitoneal injections of in house produced mAbs anti-CD4 GK1.5 (200 or 100 mg) and anti-CD8 53-6.72 (100 mg) were given 2 days before tumor engraftment and repeated every 5 days. Depletion was verified by flow cytometry on day 2 and 6 after the 1^st^ depletion and at the end of the experiment. Representative plots are shown for day 2.

**Supplementary figure S3**

**
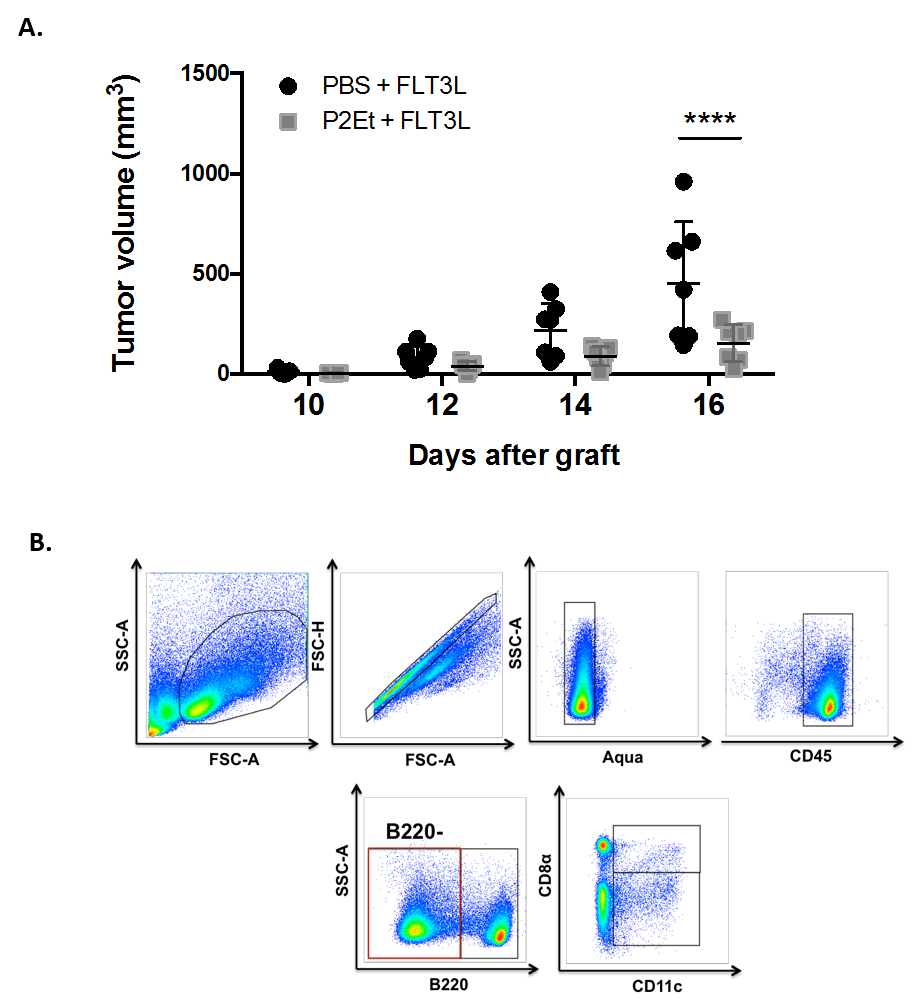
**

**Fig S3. Tumor growth and DC activation after P2Et activation in FLT3L-treated mice**. Six C57Bl/6 mice per group were engrafted with 1x10^5^ B16F10 cells and treated with P2Et (75 mg/ml) or PBS twice a week. In addition, all mice were treated with FLT3 ligand. **A**. Tumor volume was monitored. Each dot represents one individual. mean±SD is represented ****P < 0,0001 n=2. **B**. Analysis strategy for DC identification. The first gate correspond to lymphocyte gate, second to doublets exclusion, third to death cells exclusion, forth to CD45 positive cells, fifth exclude B220 cells and the final gate corresponds to CD11c+CD8a+ or CD11c+CD8- dendritic cells.

**Supplementary figure S4**

**
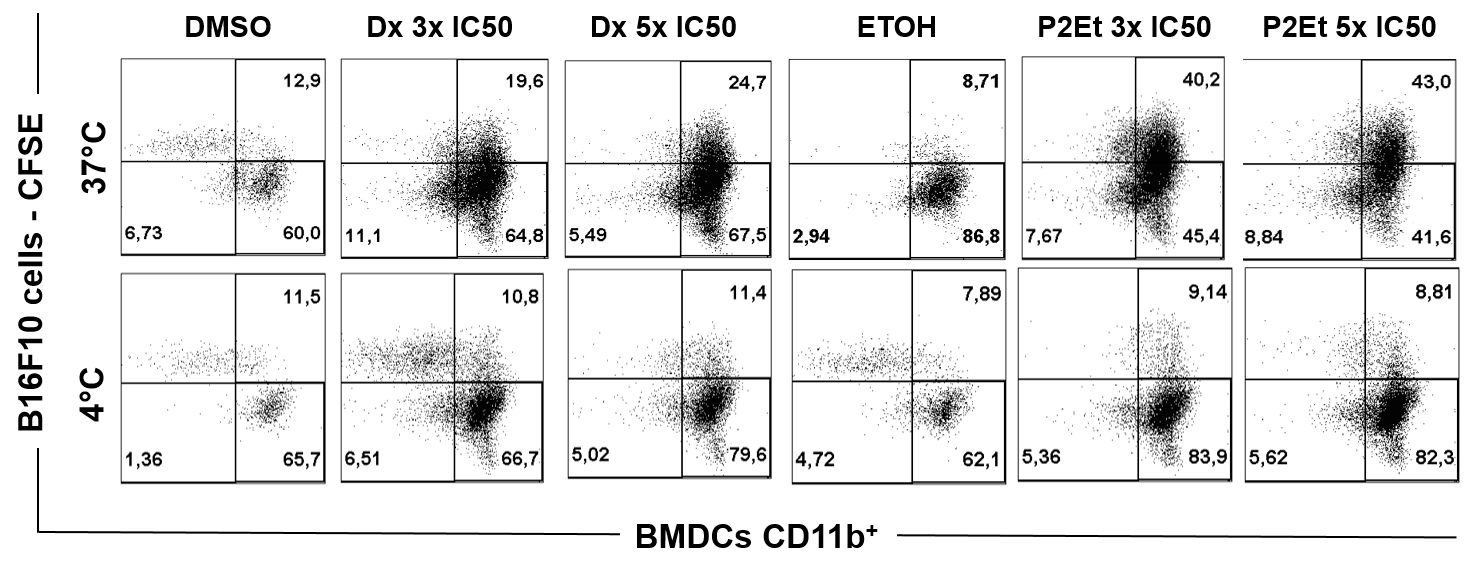
**

**Fig S4. B16F10 P2Et-treated cells induce BMDCs activation *in vitro*.** B16F10 cells were stained with CFSE and treated with P2Et or Dx. Solvents of the different treatments were used as negative controls. After 48h of treatment cells were harvested and co-cultured for 12h at 37 or 4°C with BMDCs stained with conjugated monoclonal anti-CD11b-Alexa Fluor 700 antibody. A double positive analysis was made and representative dots are shown. n=2

**Supplementary figure S5**

**
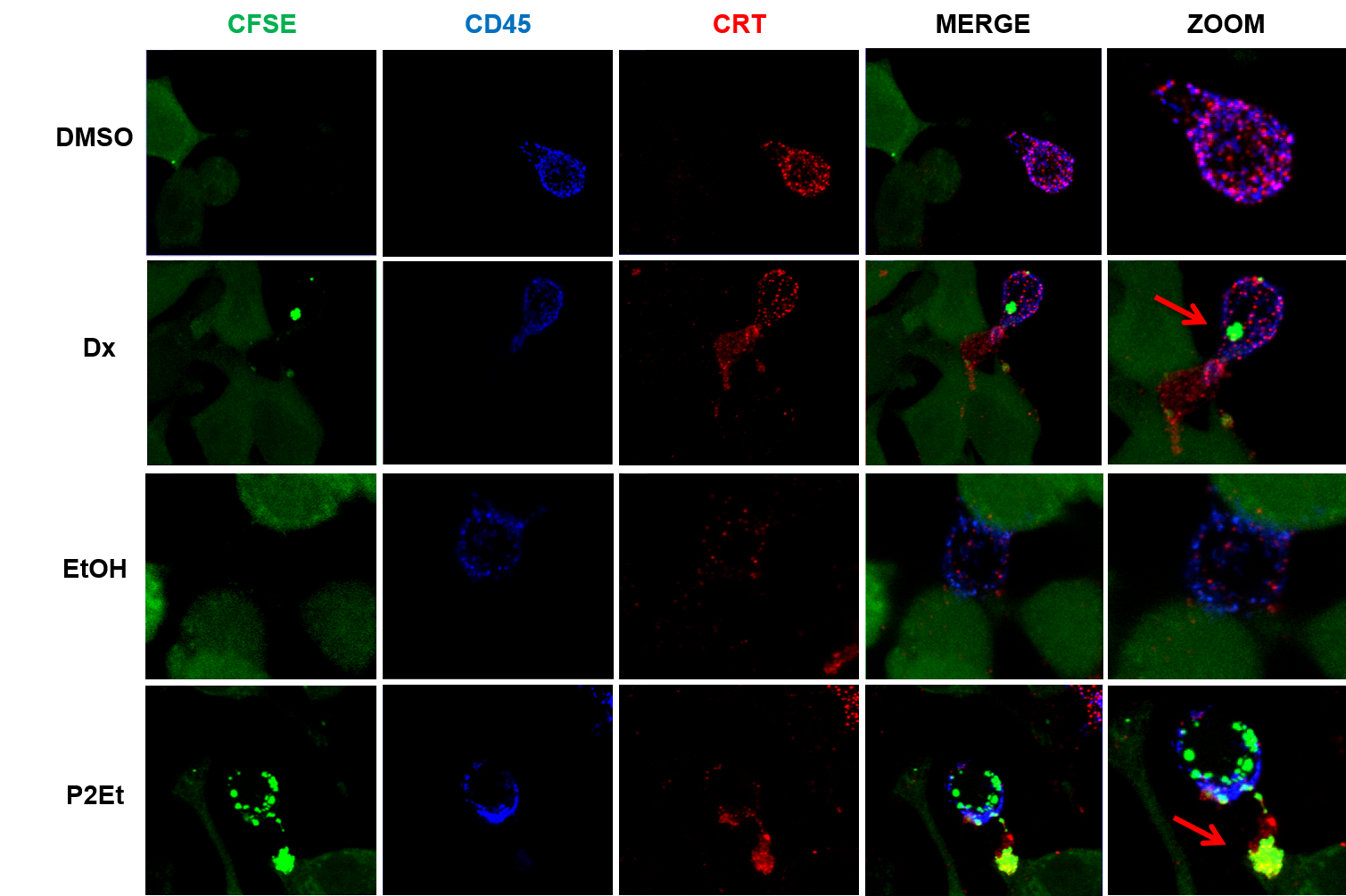
**

**Fig S5. A. Pre-treatment of B16F10 cells with P2Et induces phagocytosis by BMDCs.** B16F10 cells were stained with CFSE (Green) and treated with P2Et, Dx (IC50) or with the negative controls (ETOH, DMSO). After treatment, cells were co-cultured for 3, 8,12 and 24h with BMDCs. BMDCs were stained with the conjugated monoclonal anti-CD45-PECy5 (Blue) antibody and ecto-CRT (Red) with the primary anti-CRT polyclonal antibody followed by incubation with the conjugated anti-mouse secondary antibody (alexa fluor 594). Images were acquired with Olympus F1000 with a 60X PlanAPO objective. Representative confocal images obtained 8h after co-culture are shown (n=2).
